# Multi-Omics Integration Highlights the Role of Ubiquitination in CCl_4_-Induced Liver Fibrosis

**DOI:** 10.3390/ijms21239043

**Published:** 2020-11-27

**Authors:** Maria Mercado-Gómez, Fernando Lopitz-Otsoa, Mikel Azkargorta, Marina Serrano-Maciá, Sofia Lachiondo-Ortega, Naroa Goikoetxea-Usandizaga, Rubén Rodríguez-Agudo, David Fernández-Ramos, Maider Bizkarguenaga, Virginia Gutiérrez-de Juan, Benoît Lectez, Kerman Aloria, Jesus M. Arizmendi, Jorge Simon, Cristina Alonso, Juan J. Lozano, Matias A. Avila, Jesus M. Banales, Jose J. G. Marin, Naiara Beraza, José M. Mato, Félix Elortza, Rosa Barrio, James D. Sutherland, Ugo Mayor, María L. Martínez-Chantar, Teresa C. Delgado

**Affiliations:** 1 Liver Disease Lab, CIC bioGUNE, Basque Research and Technology Alliance (BRTA), 48160 Derio, Bizkaia, Spain; mmercado@cicbiogune.es (M.M.-G.); mserrano@cicbiogune.es (M.S.-M.); slachiondo@cicbiogune.es (S.L.-O.); ngoikoetxea@cicbiogune.es (N.G.-U.); rrodriguez@cicbiogune.es (R.R.-A.); jsimon@cicbiogune.es (J.S.); naiara.beraza@quadram.ac.uk (N.B.); 2 Precision Medicine and Metabolism Lab, CIC bioGUNE, Basque Research and Technology Alliance (BRTA), 48160 Derio, Bizkaia, Spain; flopitz@cicbiogune.es (F.L.-O.); dfernandez.ciberehd@cicbiogune.es (D.F.-R.); mbizcarguenaga@cicbiogune.es (M.B.); vgutierrez@cicbiogune.es (V.G.-d.J.); director@cicbiogune.es (J.M.M.); 3 Proteomics Platform, CIC bioGUNE, Basque Research and Technology Alliance (BRTA), CIBERehd, ProteoRed-ISCII, 48160 Derio, Bizkaia, Spain; mazkargorta@cicbiogune.es (M.A.); felortza@cicbiogune.es (F.E.); 4 Centro de Investigación Biomédica en Red de Enfermedades Hepáticas y Digestivas (CIBEREHD), Instituto de Salud Carlos III, 28029 Madrid, Spain; juanjo.lozano@ciberehd.org (J.J.L.); maavila@unav.es (M.A.A.); jesus.banales@biodonostia.org (J.M.B.); jjgmarin@usal.es (J.J.G.M.); 5 Department of Biochemistry and Molecular Biology, Faculty of Science and Technology, University of the Basque Country (UPV/EHU), 48940 Leioa, Spain; benoitlouisphilippe.lectez@ehu.eus (B.L.); jm.arizmendi@ehu.eus (J.M.A.); ugo.mayor@ehu.eus (U.M.); 6 Proteomics Core Facility-SGIKER, University of the Basque Country (UPV/EHU), 48940 Leioa, Spain; kerman.aloria@ehu.eus; 7 OWL Metabolomics, 48610 Derio, Bizkaia, Spain; calonso@owlmetabolomics.com; 8 Bioinformatics Platform, Centro de Investigación Biomédica en Red de Enfermedades Hepáticas y Digestivas (CIBEREHD), 08036 Barcelona, Spain; 9 Hepatology Programme, Center for Applied Medical Research (CIMA), IDISNA, University of Navarra, 31008 Pamplona, Spain; 10 Department of Hepatology and Gastroenterology, Biodonostia Health Research Institute, Donostia University Hospital, University of the Basque Country (UPV/EHU), 20014 San Sebastian, Spain; 11 Ikerbasque, Basque Foundation for Science, 48009 Bilbao, Bizkaia, Spain; 12 Experimental Hepatology and Drug Targeting (HEVEPHARM), IBSAL, University of Salamanca, 37007 Salamanca, Spain; 13 Ubiquitin-likes and Development Lab, CIC bioGUNE, Basque Research and Technology Alliance (BRTA), 48160 Derio, Bizkaia, Spain; rbarrio@cicbiogune.es (R.B.); jsutherland@cicbiogune.es (J.D.S.)

**Keywords:** liver fibrosis, ubiquitination, metabolomics, proliferating cell nuclear antigen (PCNA), DNA damage response (DDR)

## Abstract

Liver fibrosis is the excessive accumulation of extracellular matrix proteins that occurs in chronic liver disease. Ubiquitination is a post-translational modification that is crucial for a plethora of physiological processes. Even though the ubiquitin system has been implicated in several human diseases, the role of ubiquitination in liver fibrosis remains poorly understood. Here, multi-omics approaches were used to address this. Untargeted metabolomics showed that carbon tetrachloride (CCl_4_)-induced liver fibrosis promotes changes in the hepatic metabolome, specifically in glycerophospholipids and sphingolipids. Gene ontology analysis of public deposited gene array-based data and validation in our mouse model showed that the biological process “protein polyubiquitination” is enriched after CCl_4_-induced liver fibrosis. Finally, by using transgenic mice expressing biotinylated ubiquitin (^bio^Ub mice), the ubiquitinated proteome was isolated and characterized by mass spectrometry in order to unravel the hepatic ubiquitinated proteome fingerprint in CCl_4_-induced liver fibrosis. Under these conditions, ubiquitination appears to be involved in the regulation of cell death and survival, cell function, lipid metabolism, and DNA repair. Finally, ubiquitination of proliferating cell nuclear antigen (PCNA) is induced during CCl_4_-induced liver fibrosis and associated with the DNA damage response (DDR). Overall, hepatic ubiquitome profiling can highlight new therapeutic targets for the clinical management of liver fibrosis.

## 1. Introduction

Liver fibrosis is the excessive accumulation of extracellular matrix proteins including collagen that occurs in most types of chronic liver diseases. A recent study has shown a high prevalence of the silent liver disease, with advanced fibrosis mainly related to non-alcoholic fatty liver disease (NAFLD) in adult European subjects without known liver disease. Indeed, Caballeria et al. showed that the prevalence estimates of increased liver stiffness (≥6.8, ≥8.0, and ≥9.0 kPa) in a population-based study were 9.0%, 5.8%, and 3.6%, respectively [1]. Advanced liver fibrosis results in cirrhosis, portal hypertension, and liver failure and often requires liver transplantation. Moreover, advanced liver fibrosis and cirrhosis are also major risk factors for hepatocellular carcinoma (HCC), the third cause of cancer-related deaths worldwide [2]. During liver fibrosis, different hepatic cell types undergo specific changes as follows: hepatocytes are injured and undergo apoptosis; sinusoidal endothelial cells undergo a loss of ‘fenestrae’ that is termed capillarization of the sinusoids; the Kupffer cells, the resident macrophages of the liver, activate and produce a variety of chemokines and cytokines; lymphocytes infiltrate the injured liver and contribute to the inflammation; and finally, the quiescent hepatic stellate cells (HSCs), the main fibrogenic cell type, are activated to produce extracellular matrix proteins, a hallmark of liver fibrosis.

The post-translational modification (PTM) of ubiquitination consists of the conjugation of the small regulatory protein of ubiquitin onto substrate proteins, usually via a C-terminal glycine of ubiquitin onto a substrate lysine. This involves three main steps: activation, conjugation, and ligation, performed by ubiquitin-activating enzymes (E1s), ubiquitin-conjugating enzymes (E2s), and ubiquitin ligases (E3s), respectively. Ubiquitination can affect proteins in many ways, such as signaling for their degradation via the ubiquitin–proteasome pathway (UPS), the endolysosomal pathway, or through autophagy. Alternatively, it can alter protein cellular location, affect their activity, and promote or prevent protein interactions. In recent years, intermediates in the enzymatic machinery of ubiquitination have been shown to be altered in liver fibrosis [3]. For example, serum concentrations of free ubiquitin and multiubiquitin chains were found to be significantly higher in patients with alcoholic fibrosis [4]. Cai et al. detected reduced mRNA expression of SMAD-specific E3 ubiquitin protein ligase 2 (Smurf-2), which is a HECT domain E3 Ub ligase that ubiquitinates nuclear Smads and targets them for proteasomal degradation in a rat model of liver fibrosis [5]. Additionally, loss of Gp78, an endoplasmic reticulum (ER)-associated E3 Ub ligase, caused fibrosis in aged mice as a result of spontaneous and random ER stress [6]. Other authors have shown that the ubiquitin *C*-terminal hydrolase L1 (UCHL1) deubiquitinase is absent in quiescent HSC, but its expression is increased and positively correlates with HSC transdifferentiation in pre-clinical mouse models and clinical liver fibrosis. Importantly, pharmacological inhibition of UCHL1 in mouse models of liver fibrosis improves liver fibrosis [7]. Likewise, an increase in mRNA expression and immunoreactivity of the E3 Ub ligase synoviolin was observed in active myofibroblasts whereas fibrotic human livers also showed co-localization of synoviolin and the main fibrotic marker, alpha smooth muscle actin (α-SMA) [8]. In cell models of HSCs, the main fibrogenic cell type, it was shown that, for example, the F-box protein 31 (FBXO31) modulates its activation by promoting ubiquitination of Smad7 [9]. On the other hand, indole-3-carbinol (I3C) induces the apoptosis of HSC through receptor-interacting protein (RIP)1 K63 deubiquitination by upregulating the deubiquitinase cylindromatosis (CYLD) [10]. Even though the relevant role of specific enzymatic machinery of ubiquitination in liver fibrosis has been explored, the real impact of ubiquitination and the hepatic pattern of ubiquitination substrates under fibrosis conditions is still unknown.

High-throughput technologies have been used to enlighten many pathologies. Genomics and the availability of large array- and sequence-based data deposited in public repositories can drive complementary metabolomics and proteomics analysis to unravel the role of ubiquitination in liver fibrosis. On the other hand, difficulties in identifying the ubiquitination fingerprint in vivo are linked to the fact that ubiquitination might affect just a small fraction of any given substrate protein and most approaches lack the sensitivity to detect these small and often dynamic changes. Our group has previously used tandem ubiquitin-binding entities (TUBEs) to analyze the ubiquitination of specific targets in liver disease [11,12]. However, these are not under denaturating conditions and all the ubiquitinated reactome is isolated and not only proteins that are ubiquitinated. Here, we took advantage of a transgenic mouse expressing biotinylated ubiquitin, the bioubiquitin (^bio^Ub) mouse model [13]. By using this mouse model, we were able to specifically enrich the ubiquitinated proteome and characterize it by mass spectrometry to identify changes in the ubiquitination of target proteins as a result of carbon tetrachloride (CCl_4_)-induced liver fibrosis. We showed that ubiquitination is relevant for the pathogenesis of CCl_4_-induced liver fibrosis and several proteins are differentially ubiquitinated under these circumstances, an example being proliferating cell nuclear antigen (PCNA). Overall, ubiquitination appears to play an important role in the regulation of cell death and survival, cell function, lipid metabolism, and DNA repair during CCl_4_-induced liver fibrosis.

## 2. Results

### 2.1. Carbon Tetrachloride (CCl_4_)-Induced Liver Fibrosis: An Interplay between Injury, DNA Damage Response (DDR), and Regeneration

**Figure 1:**
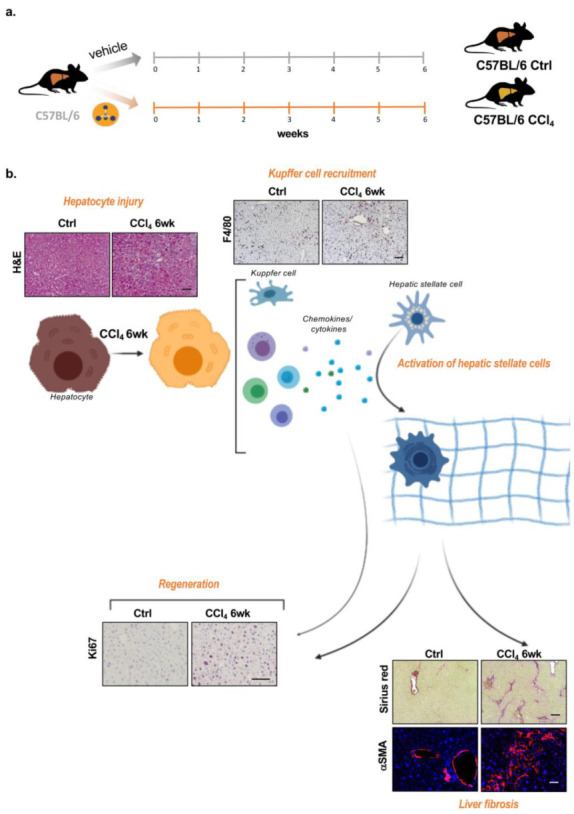
Carbon tetrachloride (CCl_4_)-induced liver fibrosis. (**a**). Schematic diagram showing the experimental protocol used to induce liver fibrosis by means of chronic intraperitoneal administration (0.6 mL/kg) of CCl_4_. (**b**). CCl_4_ induces hepatocyte damage through increased oxidative stress, which promotes the injury of cellular molecules, such as lipids, proteins, and DNA. Parenchymal disruption is observed in the hematoxylin and eosin (H&E) staining of CCl_4_-induced liver fibrosis livers. Injured hepatocytes promote the recruitment of immune system cells, such as the liver-resident macrophages, the Kupffer cells (KCs); F4/80, a membrane marker of KC is accumulated in fibrotic livers. The release of chemokines and cytokines by these immune system cells promotes the activation of hepatic stellate cells (HSCs), which, when activated, release extracellular matrix proteins, such as collagen, a hallmark of liver fibrosis but also an important intermediate in the regenerative process. Collagen fibers appear in red in Sirius red staining. Ki67 immunostaining marks proliferation/regeneration. Scale bar corresponds to 100 μm.

Carbon tetrachloride (CCl_4_) intoxication is a well-known model for producing hepatic oxidative stress and chemical injury [14]. Its biotransformation produces hepatotoxic metabolites, the highly reactive trichloromethyl free radicals, which are further converted to the peroxytrichloromethyl radicals that injure the hepatocytes. Injured hepatocytes promote the recruitment of hepatic macrophages and other cells of the immune system to produce cytokines and chemokines that cause both the proliferation of existing viable hepatocytes and, on the other hand, the activation of HSCs into myofibroblasts, the main fibrotic cell type. Activated myofibroblasts release extracellular matrix proteins, such as collagen, to further stimulate the repair process. Here, we induced liver fibrosis in wild-type animals after weekly chronic administration of CCl_4_ for 6 weeks (Figure 1a). CCl_4_-induced liver fibrosis is characterized by increased parenchymal disruption, increased recruitment of liver macrophages, the Kupffer cells, and accumulation of HSCs and collagen fibers secreted by activated HSCs. Moreover, we observed an increase in hepatocyte proliferation/regeneration markers, such as Ki67 (Figure 1b).

### 2.2. Metabolic Reprogramming in Carbon Tetrachloride (CCl_4_)-Induced Liver Fibrosis

**Figure 2:**
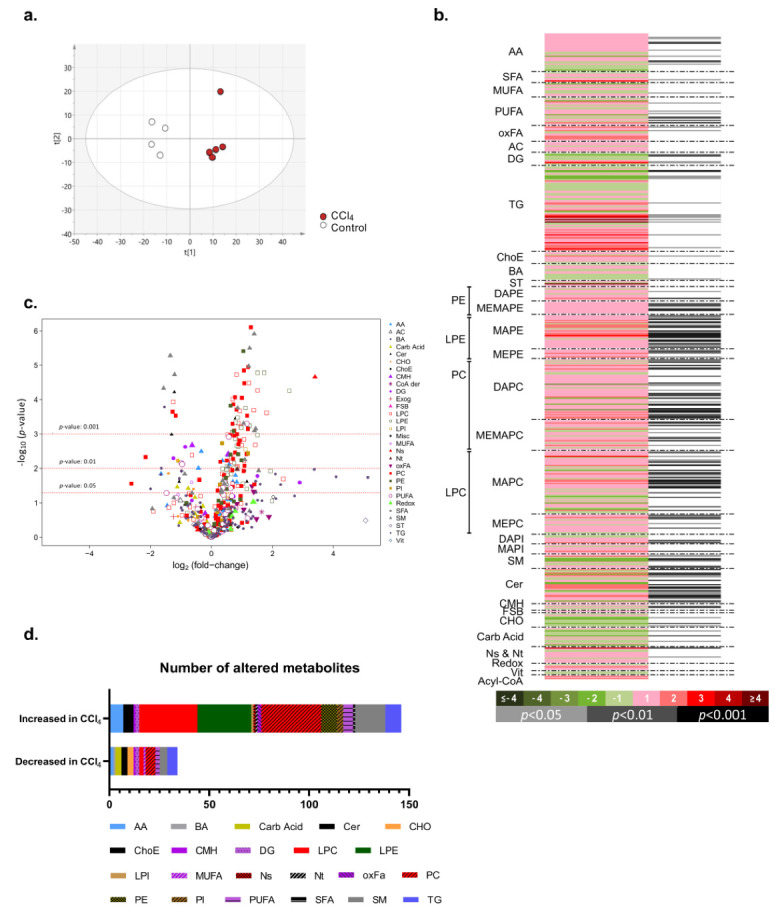
Untargeted metabolomics analysis reveals metabolic hepatic reprogramming in carbon tetrachloride (CCl_4_)-induced liver fibrosis. Three-month-old C57BL/6 wild-type animals (*n* = 4) were compared to animals treated with carbon tetrachloride (CCl_4_) once a week for 6 weeks (0.6 mL/kg) (*n* = 5). (**a**) Principal component analysis (PCA) where the data matrix is reduced to a series of principal components (PCs). A score plot was used to display each sample as situated on the projection planes described by the PCs. The first and second components explain 36.7% and 15.7% of the variability between samples, respectively. Each dot represents one sample. The ellipse represents the 95% confidence interval according to Hotelling’s T2 test. (**b**) Heat map representing the log_2_(fold change), with color code, and Student’s *t*-test (in grey scale); (**c**) Volcano plot representation showing the different fold changes and significance; and (**d**) Schematic representation of the number of increased and decreased metabolites amongst the different families of metabolites analyzed between CCl_4_-induced liver fibrosis and wild-type animals. After normality tests, metabolites were compared using the Student’s *t*-test. Abbreviations: AA. Amino acids; SFA. Saturated fatty acids; MUFA. Mono-unsaturated fatty acids; PUFA. Poly-unsaturated fatty acids; oxFA. Oxidized fatty acids; AC. Acyl carnitines; DG. Diacylglycerides; TG. Triacylglycerides; CE. cholesteryl esters; BA. Bile acids; ST. Steroids; PE. Phosphatidylethanolamines [includes diacyl-PE (DAPE) and monoacyl,monoether-PE (MEMAPE)]; LPE. Lysophosphatidylethanolamines [includes monoacyl-PE (MAPE) and monoether-PE (MEPE)]; PC. Phosphatidylcholines [includes diacyl-PC (DAPC) and monoacyl,monoether-PC (MEMAPC)]; LPC. Lysophosphatidylcholines [includes monoacyl-PC (MAPC) and monoether-PC (MEPC)]; PI. Phosphatidylinositols [includes diacyl-PI (DAPI)]; LPI. Lysophosphatidylinositols [includes monoacyl-PI (MAPI)]; SM. Sphingomyelins; Cer. Ceramides; CMH. Monohexosylceramides; FSB. Free sphingoid bases; CHO. Carbohydrates; Ns. Nucleosides; Nt. Nucleotides; Redox. Redox-electron-carriers; Vit. Vitamins; Acyl-CoA. Acetyl-coenzyme A.

In order to better understand the metabolic changes underlying CCl_4_-induced liver fibrosis, we performed wide-targeted metabolomics. The principal component analysis (PCA) shows a reprogramming of hepatic metabolism that occurs as a consequence of CCl_4_ chronic stimulus, leading to the separation in the scores plot between the two experimental groups studied (Figure 2a). Heat map and volcano plot representations indicate that CCl_4_-induced liver fibrosis was associated with augmented complexity of the metabolome, with changes in carbohydrate and in branched amino acids metabolism, as well as an important increase in some families of metabolites, such as glycerophospholipids, that form membranes and play an important role in proliferation/regeneration: phosphatidylethanolamine (PE), phosphatidylcholine (PC), lysophosphatidilethanolamines (LPE), and lysophosphatidylcholines (LPC)). Some species of ceramides and sphingolipids were also enriched after CCl_4_-induced liver fibrosis (Figure 2b–d).

### 2.3. Ubiquitination Is Aberrant in Carbon Tetrachloride (CCl_4_)-Induced Liver Fibrosis

**Figure 3:**
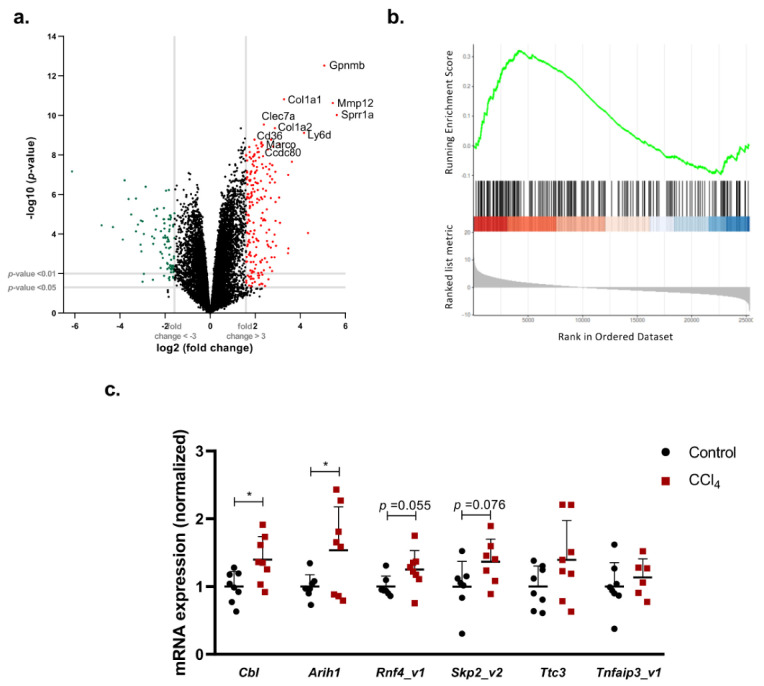
Genes related to ubiquitination are significantly dysregulated at the transcriptional level in carbon tetrachloride (CCl_4_)-induced liver fibrosis. (**a**) Volcano scatterplots showing statistical significance (*p* value) versus magnitude of change (fold change) and (**b**) polyubiqutination pathway enrichment analysis plots from a gene set enrichment analysis (GSEA) of female mice treated with CCl_4_ for 8 weeks (0.2 mL/kg/day, 3 times a week) vs. untreated mice (GSE141821). Red and green scattered points represent the most significant (* *p* < 0.01) upregulated and downregulated genes, respectively, that have a fold change higher than 3. (**c**) mRNA hepatic levels quantified by RT-PCR of genes involved in the ubiquitination pathway comparing CCl_4_-induced liver fibrosis and wild-type animals. Abbreviations: *Cbl*. Casitas B-lineage Lymphoma, *Arih1.* Ariadne RBR E3 ubiquitin protein ligase 1, *Rnf4.* Ring finger protein 4, *Skp2.* S-phase kinase-associated protein 2, *Ttc3.* Tetratricopeptide repeat domain 3, *Tnfaip3.* tumor necrosis factor alpha-induced protein 3.

Ubiquitination is a PTM that is crucial for a plethora of physiological processes. We analyzed gene array-based data deposited in Gene Expression Omnibus (GEO) of CCl_4_-induced liver fibrosis. In the gene set enrichment (GSE) data set analyzed, previously published elsewhere [15], the volcano plot shows an enrichment of the expression of genes related to activation of HSCs and accumulation of extracellular matrix proteins, such as Col1a1 (collagen, type I, alpha1) and Col1a2 (collagen, type I, alpha2), genes involved in transforming growth factor (TGFβ) signaling, such as CD63, and biomarkers of Kuppffer cells, such as the glycoprotein nonometastatic melanoma B (Gpnmb). Indeed, Gpnmb-positive macrophages have previously been shown to infiltrate in the liver during the recovery phase of CCl_4_-induced acute liver injury and contribute to the balance between fibrosis and fibrolysis in the repair process following acute liver injury [16]. Pathway enrichment analysis revealed that there is an upregulation of the genes involved in the biological processes related to protein ubiquitination (protein polyubiquitination-Gene Ontology (GO): 0000209) in liver fibrosis, suggesting that ubiquitination is relevant in the pathology of liver fibrosis (Figure 3a-b). In agreement, we confirmed that genes involved in the ubiquitination pathway are significantly upregulated in our mouse model of CCl_4_-induced liver fibrosis. Indeed, after 6 weeks of CCl_4_-induced liver fibrosis, we found increased expression of genes encoding several E3 ubiquitin-protein ligases, namely *Cbl* (Casitas B-lineage lymphoma) and *Arih1* (ariadne RBR E3 ubiquitin protein ligase 1). Other E3 ligases that are moderately increased upon CCl_4_-induced liver fibrosis include *Rnf4* (ring finger protein 4), *Skp2* (S-phase kinase-associated protein 2), *Ttc3* (tetratricopeptide repeat domain 3), and *Tnfaip3* (tumor necrosis factor alpha-induced protein 3) (Figure 3c).

### 2.4. Characterization of the Ubiquitinated Proteome in Carbon Tetrachloride (CCl_4_)-Induced Liver Fibrosis

**Figure 4:**
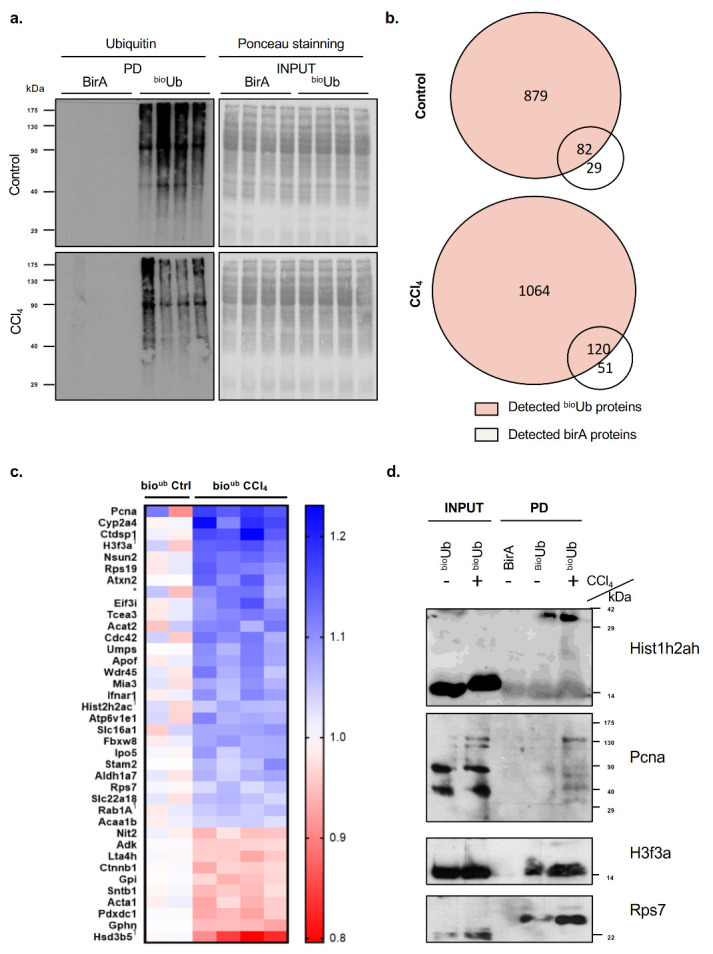
The ubiquitome fingerprint in carbon tetrachloride (CCl_4_)-induced liver fibrosis. Pulldown experiments were performed in the bioubiquitin transgenic mice (^bio^Ub) and its controls (BirA) both under control conditions and after chronic induction of liver fibrosis through weekly injection during 6 weeks of CCl_4_. (**a**) Western blot analysis of ubiquitin levels and Ponceau staining in the pulldown (PD) fraction comparing CCl_4_ animals and controls. (**b**) Venn diagram analysis comparing the total amount of identified proteins by mass spectrometry in all animals studied; (**c**) Heat map showing the significantly upregulated and downregulated ubiquitinated proteins in CCl_4_-induced liver fibrosis. * Uncharacterized protein C1orf50 homolog; (**d**) Western blot analysis validation of ubiquitinated proteins that were detected to be upregulated after CCl_4_-induced liver fibrosis by using mass spectrometry. Abbreviations: Pcna. Proliferating cell nuclear antigen; Hist1a2ah. Histone 1a2ah; H3f3a. Histone H3.3; RPS7. 40S ribosomal protein S7.

PTMs of proteins offer a fast way to regulate protein function. In order to further assess the relevance of ubiquitination and the ubiquitinated proteome in CCl_4_-induced liver fibrosis, we used ^bio^Ub mice, a transgenic mouse model expressing biotyinylated ubiquitin, and control birA mice expressing birA alone [13]. CCl_4_-induced liver fibrosis shows very similar parenchyma disruption in the ^bio^Ub mice and wild type animals (Supplementary Figure S1a). In agreement with previous evidence [17], after 6 weeks of CCl_4_ chronic administration to the ^bio^Ub and the birA mice, we detected fibrotic livers associated with parenchyma disruption and inflammation, and increased serum transaminases (Supplementary Figure S1b–e). Overall, these results show that the ^bio^Ub mice are equally susceptible to liver fibrosis. Thus, both in the ^bio^Ub and birA animals treated with CCl_4_ for 6 weeks, we characterized the ubiquitinated proteome using a pulldown assay and liquid chromatography-mass spectrometry (LC-MS) analysis. Western blots revealed an enrichment in ubiquitinated proteins in the ^bio^Ub mice relative to birA controls (Figure 4a). Venn diagrams representing the amount of identified proteins are shown in Figure 4b. For background correction, we subtracted all the proteins that were identified in the control birA mouse both in animals without stimulus or in animals stimulated with CCl_4_ for 6 weeks, respectively. A heat map shows ubiquitinated proteins that are significantly upregulated or downregulated after CCl_4_-induced liver injury in Figure 4c. In supplemental file 1 and Table S3, a complete list displaying all the proteins identified is shown. Finally, validation by Western blot was performed to confirm the mass spectrometry (MS) results for representative deregulated ubiquitinated proteins in the CCl_4_-treated mice (Figure 4d).

**Figure 5:**
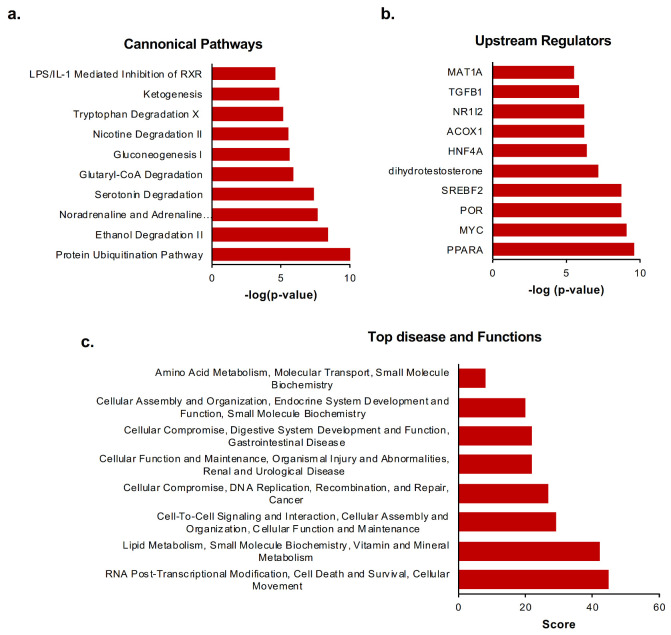
Ingenuity Pathway Analysis (IPA^®^) was performed to understand the principal pathways affected by ubiquitination after carbon tetrachloride (CCl_4_)-induced liver fibrosis. IPA analysis retrieving the (**a**) Top canonical pathways, (**b**) Upstream regulators, and (**c**) Top diseases and Functions of the differentially expressed ubiquitinated proteins identified by mass spectrometry using the bioubiquitin (^bio^Ub) and the BirA mice as a result of CCl_4_-induced liver fibrosis.

The Ingenuity Pathway Analysis software (IPA) (http://www.ingenuity.com, Ingenuity Systems., Redwood City, CA, USA) was used to identify pathways, top disease, and functions as well as canonical pathways associated with the proteins identified in the mass spectrometry analysis. Overall, ubiquitination appears to play an important role in the regulation of cell death and survival, cell function, lipid metabolism, and DNA damage repair (DDR) during CCl_4_-induced liver fibrosis. Moreover, IPA analysis retrieved a list of the top upstream regulators, where peroxisome proliferator-activated receptor alpha (PPARα) is the most significant upstream regulator altered after CCl_4_-induced liver fibrosis, followed by c-Myc (Figure 5a–c). Interestingly, a PPARα-dependent pathway DDR has been linked to events surrounding hepatocellular carcinoma, but its roles in liver fibrosis remain to be explored [18].

In summary, a ubiquitome fingerprint in CCl_4_-induced liver fibrosis was obtained for the first time by using ^bio^Ub mice combined with LC-MS analysis.

### 2.5. PCNA Ubiquitination in DNA Damage Repair (DDR) during Carbon Tetrachloride (CCl_4_)-Induced Liver Fibrosis

**Figure 6:**
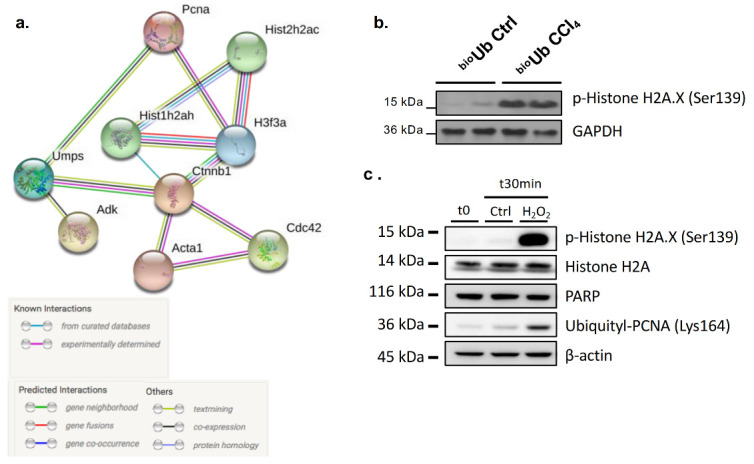
PCNA ubiquitination and DNA damage repair (DDR) during carbon tetrachloride (CCl_4_)-induced liver fibrosis and after hydrogen peroxide (H_2_O_2_)-induced injury to the HepG2 human cell line. (**a**) STRING analysis showing the major cluster of the predicted and previously demonstrated interactions between the identified ubiquitinated proteins on the mouse models of CCl_4_-induced liver fibrosis. (**b**) CCl_4_-induced liver fibrosis is associated with augmented DNA damage repair (DDR) as detected by the hyperphosphorylation of the Ser-139 residue of the histone variant H2AX, forming *γ*H2AX, a marker of activated DDR. (**c**) Stimulation of the HepG2 human cell line with H_2_O_2_ at 100 μM for 30 min results in increased *γ*H2AX and ubiquitination of PCNA at lysine 146.

Biological databases showing interacting proteins can be used to better understand the dimension of ubiquitination in CCl_4_-induced liver fibrosis. Cluster analysis of known and predicted protein–protein interactions using the STRING database [19] of the list of differential ubiquitinated proteins in CCl_4_-induced liver fibrosis showed distinct clusters of proteins. The main one involves proliferating cell nuclear antigen (Pcna); the family of histones Hist1h2ah, Hist2h2ac, and H3f3a; uridine monophosphate synthetase (Umps); adenosine kinase (Adk); catenin beta-1 (Ctnnb1); actin; alpha skeletal muscle (Acta1); and cell division control protein 42 homolog (Cdc42) (Figure 6a). PCNA is a central molecule at the crossroads of DNA replication and DNA repair, a complex molecular mechanism to detect and repair DNA damage. In agreement, after CCl_4_ stimulation, we also observed an increase in a marker of DDR, such as the hyperphosphorylation of the Ser-139 residue of the histone variant H2AX, forming γH2AX, an early marker of both single-stranded and double-stranded DNA breaks (SSDB and DSBs, respectively) (Figure 6b) [20].

Mounting evidence indicates that the ubiquitin system plays a critical role in orchestrating the major DDR pathways [21,22]. Indeed, it has been previously shown that besides K48-linked ubiquitination, PCNA undergoes a switch mechanism from a mono- to a polyubiquitylated form at position K164, regulating its activity in DNA repair [23]. In agreement, we showed that in the HepG2 hepatoma cells, stimulation with hydrogen peroxide (H_2_O_2_), previously shown to induce concentration-dependent increases in SSDB and concomitant repair in SSDB in monolayer cultures of hepatocytes [24], was associated with increased ubiquitination at lysine 164 and increased *γ*H2AX [20] (Figure 6c).

Taken together, we provide evidence that ubiquitination of proteins involved in several pathways is increased after CCl_4_-induced liver fibrosis, exemplified by the response to DNA damage and increased ubiquitination of PCNA.

## 3. Discussion

Chronic liver disease is a series of multi-factorial conditions involving the progressive destruction and regeneration of the liver parenchyma leading to liver fibrosis, which can eventually progress to cirrhosis and liver cancer. In recent years, the prevention and reversal of liver fibrosis has become the major endpoint in clinical trials to test novel liver-specific drugs. Whereas remarkable progress has been made with antiviral therapies for liver fibrosis when the underlying causes of the disease are chronic hepatitis B and C, there are still no approved treatments for the patients with advanced alcoholic or nonalcoholic steatohepatitis (NASH), and genetic or autoimmune liver diseases. A better understanding of the mechanisms involved in liver fibrosis could potentially highlight novel and druggable therapeutic targets. Omics-based techniques show a great potential to address the mechanism underlying the development of liver fibrosis.

By using wide-targeted metabolomics LC-MS-based analysis, we showed that perturbations in branched amino acids and tricarboxylic acids (TCA) cycle metabolites, along with sphingolipid and glycerophospholipid metabolites, were observed as a consequence of CCl_4_-induced liver fibrosis, one of the most common widely used animal models of liver fibrosis. These results are in agreement with previous reports showing similar findings in LC-QTOF-MS-based urinary and serum metabolic profiling of CCl_4_-treated rats [25]. In liver fibrosis and cirrhosis, both ceramide and sphingosine-1-phosphate (S1P) have been reported to be upregulated [26]. Whereas an upregulated ceramide content has been related to cell death [27], a close link has been established between sphingolipid signaling and DDR in liver disease [28,29]. Previously, it has been shown that S1P specifically binds to TNF receptor-associated factor 2 (TRAF2) at the amino-terminal RING domain and stimulates its E3 ligase activity [30], suggesting that metabolic deregulation of sphingolipid metabolism could play a role in the regulation of ubiquitination. Furthermore, analysis of genomic data shows increased expression of genes involved in ubiquitination observed in CCl_4_-induced liver fibrosis, both by using repository data and further validation in our mouse model of CCl_4_-induced liver fibrosis. Of relevance, one of the ubiquitin ligases overexpressed in CCl_4_-induced liver fibrosis, ARIH1, has been previously shown to be a potent mediator of DNA damage-induced translation arrest that protects stem and cancer cells against genotoxic stress [31]. While these data suggested that changes in ubiquitination might occur, the in vivo ubiquitome fingerprint in liver fibrosis had not been previously addressed.

Ubiquitome profiling is rather challenging, mostly because only a small portion of the total protein is modified by ubiquitin. It is further complicated by the difficulty in distinguishing between covalent modifications by ubiquitin versus non-covalent interactors of ubiquitin, as well as by the presence of highly active deubiquitinases that act during cell lysis. To address these issues, Lectez and colleagues generated a transgenic mice ideal for ubiquitin proteomics, the transgenic ^bio^Ub mice [13]. This transgenic mouse model produces a biotynilated ubiquitin in vivo and takes advantage of the biotin–avidin interaction, denaturing lysis conditions and stringent washes to ensure that only proteins conjugated directly to ubiquitin are isolated. The ^bio^Ub transgenic mouse further allows changes in the ubiquitination profile to be identified under different conditions, such as, for example, during liver injury. For the first time to our knowledge, we present here the hepatic ubiquitome profile of CCl_4_-induced liver fibrosis derived from the in vivo mouse model.

IPA analysis, using as input the differentially ubiquitinated proteins after CCl_4_-induced liver fibrosis, shows that DNA repair is one of the most altered pathways. In agreement, we showed that during CCl_4_-induced liver fibrosis, an increase in the activation of DDR is observed. These results are in agreement with very recent data from Akazawa et al. showing that the marker of the DNA damage response, the p53-binding protein 1 (53BP1), is associated with disease progression in patients with non-alcoholic fatty liver disease (NAFLD), particularly in those with more severe disease characterized by liver fibrosis [32]. During liver fibrosis, multiple metabolic changes affecting different hepatic cells occur, considering that hepatocyte injury is usually considered the triggering event in liver fibrosis. Upon injury, these cells may undergo cell death or alternatively initiate a regenerative process. For example, during acetaminophen-induced liver injury, Borude et al. showed that a lack of prompt DNA DSB repair after acetaminophen overdose leads to prolonged growth arrest and proliferative senescence, resulting in inhibited liver regeneration, establishing a clear link between DDR and regeneration in liver injury [33]. Therefore, we hypothesize that increased DDR during liver fibrosis favors the regenerative process.

STRING analysis of known and predicted protein–protein interactions, using as input the differentially expressed ubiquitinated proteins in CCl_4_-induced liver fibrosis, showed an important cluster formed by Pcna, the most differentially ubiquitinated protein after CCl_4_-induced liver fibrosis. PCNA is a central molecule at the crossroads of DNA replication and DNA repair. Previous work has shown that application of H_2_O_2_ to monolayer cultures of hepatocytes caused concentration-dependent increases in SSDB, and concomitant repair in SSDB occurred [24]. In agreement, the addition of H_2_O_2_ to HepG2 cells results in hyperphosphorylation of the Ser-139 residue of the histone variant H2AX, forming *γ*H2AX, a marker of activated DDR [20], in association with augmented PCNA ubiquitination at position K164. These results agree with early evidence showing that during DNA repair, PCNA undergoes a switch mechanism from a mono- to a polyubiquitylated form at position [23].

Other ubiquitinated proteins identified by LC-MS and differentially expressed in CCl_4_-induced liver fibrosis that cluster with PCNA are two histone families, histone H2a type 1-H (Hist1h2ah, Hist2h2ac) and histone H3.3 (H3f3a). It is known that DDR pathways have evolved to sense, signal, and repair DNA damage within the chromatin environment. Histone proteins, which constitute the building blocks of chromatin, are highly modified by PTMs that regulate chromatin structure and function. Histone mono-ubiquitination has emerged as a key player in cellular response to DNA damage [34]. Indeed, histone H2A was the first protein that was identified to be ubiquitinated [35]. Indeed, dynamic regulation of RNF168-mediated ubiquitination of histone H2A Lys13,15 (H2AK13,15ub) at DSBs is crucial for preventing aberrant DNA repair and maintaining genome stability [36]. Additionally, other authors have shown that the E3 ubiquitin ligase NEDD4 ubiquitinates histone H3 on lysine 23/36/37 residues, which specifically recruits histone acetyltransferase GCN5 for subsequent H3 acetylation, creating a chromatin micro-environment that is rich in *γ*H2AX and acetylated H3 [37].

In summary, by using multi-omics approaches together with the transgenic ^bio^Ub mouse and LC-MS analysis, we have shown for the first time the increase of ubiquitination, together with the ubiquitinated proteome signature associated with CCl_4_-induced liver fibrosis. Moreover, we documented metabolic changes, as well as changes in the expression of genes encoding ubiquitin-related enzymatic machinery, in fibrosis-inducing conditions. Of the proteins with enhanced ubiquitination, PCNA and other DDR components are of key interest. Further studies are necessary to understand how ubiquitination can account for failure in DDR and the progression towards hepatocarcinogenesis brought on by liver fibrosis, keeping in mind that targeting ubiquitination of proteins, such as PCNA or histones, could pave the way for therapeutic approaches that could halt disease progression in liver fibrosis.

## 4. Materials and Methods

### 4.1. Mouse Models

All animal procedures were performed at the AAALAC–accredited CICbioGUNE animal facility under approved protocols from the CIC bioGUNE Institutional Animal Care and Use Committee and the Country Council of Bizkaia (P0918, 2018). Three-month-old male wild-type (WT) C57BL/6 mice were acquired from Charles River (St Germain sur l’Abresie, France) and previously described transgenic male mice expressing biotinylated ubiquitin (BioUb) and their controls (BirA) [13] were bred at our CICbioGUNE animal facility. Animals were maintained on a 12/12-h light/dark cycle at 21 ± 1 °C, humidity of 45 ± 10%, and had ad libitum access to water and standard chow diet (Teklad Global 14% Protein Rodent Maintenance diet; Envigo RMS Spain, Sant Feliu de Codines, Spain).

### 4.2. Carbon Tetrachloride (CCl_4_)-Induced Liver Fibrosis

Carbon tetrachloride (CCl_4_) (Sigma-Aldrich, St. Quentin Fallavier (Lyon), France) was intraperitoneally injected in mice (0.6 mL/kg) once a week for 6 weeks in order to induce liver fibrosis and a vehicle was used for control mice. After 6 weeks of treatment, mice were sacrificed, blood withdrawn, and liver samples were immediately frozen at −80 °C. Serum samples were analyzed for transaminases using a Selectra Junior Spinlab 100 analyser (Vital Scientific, Dieren, Netherland) according to the manufacturer’s suggested protocol.

### 4.3. Histology Analysis

Hematoxylin and eosin staining together with Sirius Red staining and F4/80 immunohistochemistry were performed as previously described [38].

### 4.4. Metabolomics Analysis by Liquid Chromatography-Mass Spectrometry (LC-MS) Analysis

Metabolomics analysis was performed by OWL Metabolomics (Bizkaia, Spain). Metabolite extraction was accomplished by fractionating the liver samples into pools of species with similar physicochemical properties, using appropriate combinations of organic solvents. A different LC-MS method was used for the analysis of each extract: Two separate LC-time of flight (ToF)-MS-based platforms that analyzed methanol (platform 1) and methanol/chloroform (platform 2) extracts for lipid analyses were combined with a LC-single quadrupole-MS amino acid analysis (platform 3) and a LC-ToF-MS platform for the analysis of polar metabolites in a methanol/H_2_O extract (platform 4) [12,39]. Identified metabolites in the methanol extract platform included fatty acids, acyl carnitines, bile acids, lysoglycerophospholipids, free sphingoid bases, and oxidized fatty acids. The chloroform/methanol extract platform provided coverage over glycerolipids, cholesterol esters, sphingolipids, and glycerophospholipids. 

Briefly, proteins were precipitated by adding H_2_O (15:1, *v*/*w*), methanol containing the internal standards used for platforms 1 and 4 (50:1, *v*/*w*), chloroform:methanol (2:1) containing internal standards used for platform 2 (4:1, *v*/*w*), and chloroform (40:1, *v*/*w*) to the liver tissue (15 mg). The resulting mixture was homogenized using a Precellys 24 homogenizer (Bertin Technologies, Montigny-le-Bretonneux, France) at 6500 rpm for 45 s × 1 round. Homogenized samples were incubated at −20 °C for 1 h and after vortexing, 500 µL were collected for platforms 1, 2, and 4. For platform 1, supernatants were centrifuged, dried under vacuum, and reconstituted in methanol. For platform 2, the supernatants were mixed with H_2_O and incubated for 1 h at −20 °C. After centrifugation, the organic phase was collected and dried under vacuum. Dried extracts were reconstituted in acetonitrile/isopropanol (1:1) for LC-MS analysis. For platform 3, 10-μL aliquots from the extracts prepared for platform 1 were derivatized for amino acid analysis. For platform 4, supernatants were mixed with chloroform and H_2_O. After centrifugation, extracts were dried under vacuum and reconstituted in H_2_O.

A test mixture of standard compounds was analyzed before and after the entire set of randomized duplicated sample injections in order to examine the retention time stability (generally < 6-s variation, injection-to-injection), mass accuracy, and sensitivity of the system throughout the course of the run, which lasted a maximum of 34 h per batch of samples injected. For each injection batch, the overall quality of the analysis procedure was monitored using five repeat extracts of the QC Validation sample. Peak detection, noise reduction, and data normalization steps were performed as previously described [40].

### 4.5. Transcriptomics Analysis

The raw gene expression data from 1 dataset was downloaded from the GEO data repository (GSE141821) [15]. Dataset was processed using bioconductor tools [41]. Briefly, we used the entrez-based probe definition [42], normalization using rma [43], and differential expressed ranking using moderated *t*-statistics [44]. Gene Set Enrichment Analysis (GSEA) was done using gseGO funtion from clusterProfiler [45].

### 4.6. Real-Time Polymerase-Chain Reactions (RT-PCR)

Total RNA of cells was isolated with a TRIzol (Invitrogen, van Allen Way, Carlsbad CA; USA) and quantitative RT-PCR was performed as previously described [38]. Primers sequences are described in Table S1.

### 4.7. Pulldown Assay

First of all, livers were homogenized using 1 mL of Lysis Buffer (1× PBS, 8M Urea, 1% SDS) per 250 mg of tissue. Subsequently, it was centrifuged at 14,000 rpm for 5 min at 4 °C, pellet was discarded, and 50 µL of supernatant were kept for use as input for Western blots. High Capacity Neutravidin (Thermo Fisher Scientific, Waltham, MA, USA) required the removal of chemical preservatives using 0.05% Tween-PBS and an incubation with filtered Binding Buffer (1× PBS, 3.15 M Urea, 0.25% SDS, 1 M NaCl) at room temperature for 5 min with constant inversion. Supernatant of the liver homogenate was also mixed with same volume of Binding Buffer. Then, 250 µL of pretreated Neutravidin beads were added to the supernatant mix and incubated for 3 h at room temperature with constant inversion. After, samples were centrifuged at 2000 rpm for 5 min and 50 µL of supernatant were recovered and kept for use as flow through for Western blots. Next, bioubiquitinated proteins attached to Neutravidin beads were washed with different filtered buffers in a consecutive manner: 2 washes with Buffer 1 (1× PBS, 8.4M Urea, 0.25% SDS); 3 washes with Buffer 2 (1× PBS, 6M Gdn-HCl); 1 wash with Buffer 3 (1× PBS, 6.3M Urea, 1M NaCl, 0.2% SDS); 3 washes with Buffer 4 (1× PBS, 4.2M Urea, 1M NaCl, 10% Isopropanol, 10% EtOH, 0.2% SDS); 1 wash with Buffer 1; 1 wash with Lysis Buffer; and 3 washes with Buffer 5 (1× PBS, 2% SDS).

### 4.8. Proteomics Analysis by LC–MS/MS Analysis

Protein samples were boiled for 5 min and resolved in 12.5% acrylamide gels, using a Mini-Protean II electrophoresis cell (Bio-Rad, Hercules, CA, USA). A constant voltage of 150 V was applied for 45 min. Gels were fixed in a solution containing 10% acetic acid, 40% ethanol for 30 min, and stained overnight in Coomassie Brilliant Blue (USB Corporation, Cleveland, OH, USA). Gels were then washed in a solution containing 30% ethanol and 10% acetic acid for 30 min, and the gel was cut according to the distribution of the signal. Each lane was subjected to tryptic digestion, followed by LC–MS/MS analysis.

Gel bands were sliced into 5 small pieces as accurately as possible to guarantee reproducibility. The slices were subsequently washed in milli-Q water. Reduction and alkylation were performed using ditiothreitol (10 mM DTT in 50 mM ammonium bicarbonate) at 56 °C for 20 min, followed by chloroacetamide (50 mM chloroacetamide in 50 mM ammonium bicarbonate) for another 20 min in the dark. Gel pieces were dried and incubated with trypsin (12.5 µg/mL in 50 mM ammonium bicarbonate) for 20 min on ice. After rehydration, the trypsin supernatant was discarded. Gel pieces were hydrated with 50 mM ammonium bicarbonate, and incubated overnight at 37 °C. After digestion, acidic peptides were cleaned with TFA 0.1% and dried out in a RVC2 25 speedvac concentrator (Christ, Osterode am Harz, Germany). Peptides were resuspended in 10 µL 0.1% FA and sonicated for 5 min prior to analysis.

LC-MS/MS analysis of peptides was carried out using a nanoUPLC chromatography system (EASY-nLC 1000, Thermo Fisher Scientific, Waltham, MA, USA) interfaced with a Q Exactive mass spectrometer (Thermo Fischer Scientific, Waltham, MA, USA)via a Nanospray Flex ion source (Thermo Fisher Scientific, Waltham, MA, USA) Peptides were loaded onto an Acclaim PepMap100 pre-column (75 µm × 2 cm, ThermoFisher Scientific, Waltham, MA, USA) connected to an Acclaim PepMap RSLC (50 µm × 15 cm, Thermo Scientific, Waltham, MA, USA) analytical column. Peptides were eluted from the column using a linear gradient of 3 to 30% acetonitrile in 0.1% formic acid at a flow rate of 300 nL min^−1^ over 60 min. Q Exactive was operated in a top 10 data-dependent mode. Survey scans were acquired at a resolution of 70,000 (*m*/*z* 200) and fragmentation spectra at 17,500 (*m*/*z* 200). Precursors were fragmented by higher energy C-trap dissociation (HCD) with normalized collision energy of 28 eV. The maximum injection time was 120 ms for both, survey and MS/MS scan, whereas AGC target values of 3e6 and 5e5 were used for survey and MS/MS scans, respectively. Repeat sequencing of the peptide was minimized by excluding the selected peptide candidates for 30 s. Singly charged ions and ions with an unassigned charge state were also excluded from MS/MS.

For protein and ubiquitylated peptide identification, raw data files were searched against the UniProtKB Swiss-Prot mouse database v2014.05 (24,674 mouse sequence entries + TRYP_PIG sequence) using Proteome Discoverer 1.4 (Thermo Fisher Scientific, Waltham, Massachusetts, USA) Precursor and fragment mass tolerances were set to 10 ppm and 0.05 Da, respectively. Enzyme specificity was set to trypsin, with a maximum of 2 missed cleavages. Carbamidomethylation of C was set as fixed modification whereas oxidation of M, and GlyGly and LeuArgGlyGly of K were selected as variable modifications for database searching. The false discovery rate was set at 0.01 at the PSM level. In addition, MaxQuant proteomics computational platform v1.5.3.17 was used for the differential analysis. Searches were done against a UniProtKB Swiss_Prot mouse database (v2015.09, 24762 sequence entries) using the Andromeda search engine [46]. Carbamidomethylation (C) was set as fixed modification whereas M oxidation, protein *N*-terminal acetylation, and K GlyGly were defined as variable modifications. Mass tolerance was set to 8 and 20 ppm at the MS and MS/MS level, respectively. Enzyme specificity was set to trypsin, allowing *N*-terminal to P cleavage, with a maximum of 1 missed cleavage. Match between runs option was enabled with a 1.5 min match time window and 20 min alignment window to match identification across samples. The minimum peptide length was set to 7 amino acids. The false discovery rate for peptides and proteins was set to 1%. Normalized spectral protein intensities [Label free-quantification (LFQ intensity)] were calculated using the MaxLFQ algorithm. MaxQuant output data were analyzed with the Perseus module (v1.5.2.6) [47]. Potential contaminants, reverse hits, and proteins identified with a single modified peptide were removed. Missing LFQ intensity values were replaced using the replace missing values from a normal distribution option in Perseus (width 0.3 and down shift 1.8). To determine statistically significant changes in protein abundance, the two-tailed Student’s *t* test was used. Proteins displaying a *p*-value smaller than 0.05 and a LFQ fold change bigger or smaller than 2 were considered as differentially expressed proteins

### 4.9. Western Blotting

Following protein quantification and normalization, whole-cell extracts were fractionated by SDS-PAGE and transferred to a polyvinyldene difluride membrane. After incubation with 5% nonfat milk in tris-buffered saline 0.1% Tween^®^20 detergent (TBST) (10mM Tris, pH 8.0, 150mM NaCl, 0.5% Tween 20) for 60min, the membrane was incubated with primary antibodies at 4 °C for 16 h (Table S2). Membranes were washed three times for 10 min and incubated with a 1:5000 dilution of horseradish peroxidase-conjugated anti-mouse or anti-rabbit antibodies for 1 h. Blots were washed again with TBST and developed with the ECL system (Bio-Rad, Hercules, CA, USA).

### 4.10. HepG2 Cell Line Treatments

HepG2 cells were acquired from ATCC^®^. Cells were maintained at 37 °C and 5% CO_2_ in 10% fetal bovine serum (FBS; GIBCO, Thermo Fisher Scientific, Waltham, Massachusetts, USA) Dulbecco’s Modified Eagle Medium (DMEM; GIBCO, Thermo Fisher Scientific, Waltham, Massachusetts, USA) containing penicillin, streptomycin and amphotericin B (100 U/mL), and glutamine (2 mM). Six hours before starting the experiment, medium was changed into 0% FBS DMEM. Cells were treated with 100 µM hydrogen peroxide (H_2_O_2_) and compared to no treatment conditions. After 30 min, cell extracts were obtained using RIPA buffer and sonication before centrifugation (14,000 rpm for 30 min at 4 °C). Supernatants were frozen at −80 °C until further analysis.

### 4.11. Statistical Analysis

The results are reported as mean ± standard error of the mean (SEM). Statistical analysis was performed using Student’s *t*-test as appropriate. Statistical analyses were done by PRISM software v5 (GraphPad, San Diego, CA, USA). A *p* value < 0.05 was considered statistically significant.

## Supplementary Materials

The supplementary materials are available online at https://www.mdpi.com/1422-0067/21/23/9043/s1.

## Abbreviations

α-SMA—Alpha smooth muscle actin; AA—Amino acids; AC—Acyl carnitines; Acta1—Alpha skeletal muscle; Acyl-CoA—Acetyl-coenzyme A; Adk—Adenosine kinase; Arih1—Aariadne RBR E3 ubiquitin protein ligase 1; BA—Bile acids; bioUb—Biotinylated ubiquitin; C—Carbamidomethylation; Cbl—Casitas B-lineage lymphoma; CCl_4_—Carbon tetrachloride; Cdc42—Cell division control protein 42 homolog; CE—cholesteryl esters; Cer—Ceramides; CHO—Carbohydrates; CMH—Monohexosylceramides; Col1a1—collagen, type I, alpha1; Col1a2—collagen, type I, alpha2; Ctnnb1—Catenin beta-1; CYLD—Cylindromatosis; DDR—DNA damage response; DG—Diacylglycerides; DSB—Double-stranded DNA breaks; ER—Endoplasmic reticulum; FBXO31—F-box protein 31; FSB—Free sphingoid bases; GEO—Gene Expression Omnibus; GO—Gene Ontology; Gpnmb—Glycoprotein nonometastatic melanoma B; GSE—Gene set enrichment; GSEA—Gene set enrichment analysis; I3C—Indole-3-carbinol; H&E—Hematoxylin and eosin; H2O2—Hydrogen peroxide; HCC—Hepatocellular carcinoma; HCD—Higer energy C-trap dissociation; Hist1a2ah—Histone 1a2ah; H3f3a—Histone H3.3; HSCs—Hepatic stellate cells; IPA—Ingenuity Pathway Analysis; KCs—Kupffer cells; LC-QTOF-MS—Liquid chromatography -quadrupole time of flight- mass spectrometry; LC-MS—Liquid chromatography- mass spectrometry; LFQ—Label free-quantification; LPC—lysophosphatidylcholines; LPE—Lysophosphatidilethanolamines; LPI—Lysophosphatidylinositols; MUFA—Mono-unsaturated fatty acids; MS—Mass spectrometry; NAFLD—Non-alcoholic fatty liver disease; NASH—Non-alcoholic steatohepatitis; Ns—Nucleosides; Nt—Nucleotides; oxFA—Oxidized fatty acids; PPARα—Peroxisome proliferator-activated receptor alpha; PCA—Principal component analysis; PCNA—Proliferating cell nuclear antigen; PC—Phosphatidylcholine; PCs—Principal components; PE—Phosphatidylethanolamine; PI—Phosphatidylinositols; PTM—Post-translational modification; PUFA—Poly-unsaturated fatty acids; Redox—Redox-electron-carriers; RIP—Receptor-interacting protein; Rnf4—Ring finger protein 4; RPS7—40S ribosomal protein S7; S1P—Sphingosine-1-phosphate; SEM—Standard error of the mean; SFA—Saturated fatty acids; Skp2—S-phase kinase-associated protein 2; SM—Sphingomyelins; Smurf-2—SMAD-specific E3 ubiquitin protein ligase 2; SSDB—Single-stranded DNA breaks; ST—Steroids; TBST—Tris-buffered saline 0.1% Tween®20 detergent; TCA—Tricarboxylic acids; TG—Triacylglycerides; TGFβ—Transforming growth factor; Tnfaip3—Tumor necrosis factor alpha-induced protein 3; TRAF2—TNF receptor-associated factor 2; Ttc3—Tetratricopeptide repeat domain 3; TUBEs—Tandem ubiquitin-binding entities; UCHL1—Ubiquitin C-terminal hydrolase L1; Umps—Uridine monophosphate synthetase; UPS—Ubiquitin-proteasome pathway; Vit—Vitamins; WT—Wild-type.
