# Supplementary material for: Multi-Omics Integration Highlights the Role of Ubiquitination in CCl4-Induced Liver Fibrosis
Source: Int J Mol Sci. 2020 Nov 27;21(23):9043. doi: 10.3390/ijms21239043 (PMC7729774; doi:10.3390/ijms21239043)
Supplement: Supplementary file 1 [file ijms-21-09043-s001.pdf]

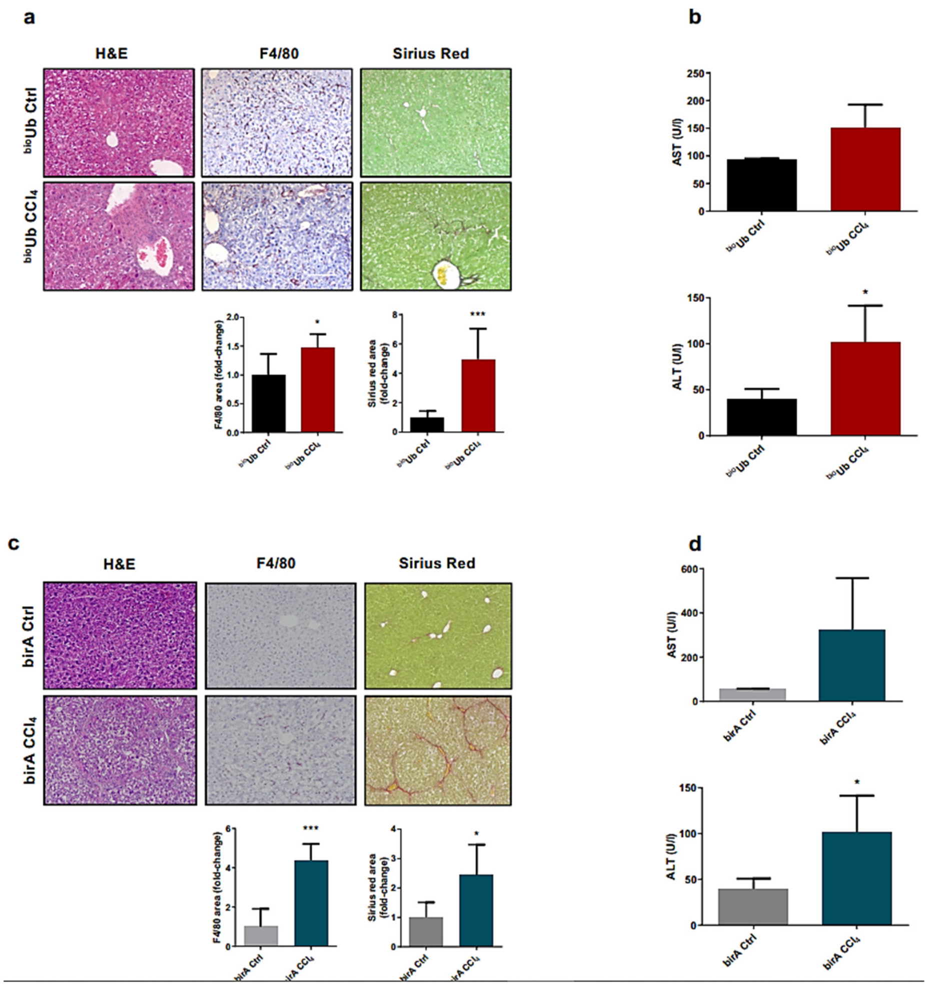

Figure S1. Histological and serological characterization of the biobiquitin ([bio]Ub) and birA mice after carbon tetrachloride (CCl_4)-induced liver fibrosis. (**a**) Hematoxylin & eosin (H&E) staining in the [bio]Ub mice and wild type animals under basal conditions and after CCl_4-induced liver fibrosis. Hematoxylin & eosin (H&E) staining, immunostaining of F4/80 macrophage marker, sirius red staining, and respective quantifications, as well as serum transaminases in (**b**) and (**c**) bioubiquitin ([bio]Ub) mice, control (n = 5) and after CCl_4 administration (n = 5); and (**d**) and (**e**) birA mice, control (n = 5) and after CCl_4 administration (n = 5). Data are represented as average ± SEM, *p <0.05 is represented.

Table S1. Primers sequences used in Real-Time PCR. Casitas B-lineage lymphoma (Cbl), Adriane RBR E3 Ubiquitin Protein Ligase 1 (Arih1), Ring finger protein 4 version 1 (Rnf4_v1), S-phase kinase-associated protein 2 version 2 (Skp2_v2), Tetratricopeptide Repeat Domain 3 (Ttc3), Tumor necrosis factor, alpha-induced protein 3 version 1 (Tnfaip3_v1).

Gene	Primer
Cbl	F: 5'- AGTGCTGGAAGCTCATGGAC
	R: 5'- GAGGTGCTGGTAGGTGTCAG
Arih1	F: 5'- TTCAGGTTGCCCAGAGGGAA
	R: 5'- GTGGCAGTTACCCACCTAAGTT
Rnf4_v1	F: 5'- CTGGAGTCGGTACGTCCTTG
	R: 5'- GGAGCCAACTAGATGTGCAGT
Skp2_v2	F: 5'- CGGTGTTTCCTCTGGAACGA
	R: 5'- TAACACTTCGTGGCTGTCCC
Ttc3	F: 5'- GAGGGAGGTCTCAGTTTGGC
	R: 5'- ATTGCATACCCACGCCTTCA
Tnfaip3_v1	F: 5'- GGAACTGCCCAGTCTGTAGT
	R: 5'- GGGTGTGCACGTCTTTCGG

Table 2. Antibodies used in Western Blot reaction. PARP (poly (ADP-ribose) polymerase), PCNA (proliferating cells nuclear antigen).

Antibody	Host	Dilution	Vendor
p-Histone H2A.X	Rabbit	1:1000	Abcam, GB, product no. ab81299
Histone H2A	Rabbit	1:1000	Abcam, GB, product no. ab88770
PARP	Rabbit	1:1000	Cell Signaling Technology, USA, product no. 9542
PCNA	Mouse	1:1000	Santa Cruz Biotechnology, USA, product no. sc-56
Ubiquityl-PCNA (Lys 164)	Rabbit	1:1000	Cell Signaling Technology, USA, product no. 13439
beta-actin	Mouse	1:1000	Sigma-Aldrich, USA, product no. A5441
anti-rabbit	Horse	1:1000	Cell Signaling Technology, USA, product no. 7074S
anti-mouse	Horse	1:1000	Cell Signaling Technology, USA, product no. 7076S

Table 3. Differential ubiquitinated proteins identified by LC-MS proteomics analysis.

Accession	T: Protein names	Gene name	p-value	Ratio CCl_4/Ctrl
Q8CGP6	Histone H2A type 1-H;Histone H2A.J;Histone H2A type 1-K;Histone H2A type 1-F;Histone H2A type 3;Histone H2A type 1	Hist1h2ah;H2afj;Hist1h2ak;Hist1h2af;Hist3h2a;Hist1h2ab	1,7E-03	344,17
P17918	Proliferating cell nuclear antigen	Pcna	4,2E-02	16,00
P15392	Cytochrome P450 2A4	Cyp2a4	9,0E-03	14,82
P58466	Carboxy-terminal domain RNA polymerase II polypeptide A small phosphatase 1	Ctdsp1	4,1E-03	13,95
P84244	Histone H3.3;Histone H3.2;Histone H3.1;Histone H3.3C	H3f3a;Hist1h3b;Hist1h3a;H3f3c	7,5E-03	11,66
Q1HFZ0	tRNA (cytosine(34)-C(5))-methyltransferase	Nsun2	8,0E-04	7,29
Q9CZX8	40S ribosomal protein S19	Rps19	3,8E-03	6,81
O70305	Ataxin-2	Atxn2	7,4E-03	6,41
Q5EBG8	Uncharacterized protein C1orf50 homolog		5,0E-02	5,86
Q9QZD9	Eukaryotic translation initiation factor 3 subunit I	Eif3i	1,4E-02	5,82
P23881	Transcription elongation factor A protein 3	Tcea3	1,9E-03	5,69
Q8CAY6	Acetyl-CoA acetyltransferase, cytosolic	Acat2	4,2E-02	5,62
P60766	Cell division control protein 42 homolog	Cdc42	2,8E-02	5,43
P13439	Uridine 5-monophosphate synthase	Umps	5,2E-03	4,53
Q91V80	Apolipoprotein F	Apof	4,5E-03	4,38
Q91VM3	WD repeat domain phosphoinositide-interacting protein 4	Wdr45	3,2E-02	4,28
Q8BI84	Melanoma inhibitory activity protein 3	Mia3	1,6E-02	4,11
P33896	Interferon alpha/beta receptor 1	Ifnar1	1,0E-02	4,10
Q64523	Histone H2A type 2-C;Histone H2A type 2-A	Hist2h2ac;Hist2h2aa1	4,4E-02	4,00
P50518	V-type proton ATPase subunit E 1	Atp6v1e1	3,6E-02	3,98
P53986	Monocarboxylate transporter 1	Slc16a1	2,7E-02	3,87
Q8BIA4	F-box/WD repeat-containing protein 8	Fbxw8	2,7E-03	3,39
Q8BKC5	Importin-5	Ipo5	1,2E-02	3,26
O88811	Signal transducing adapter molecule 2	Stam2	4,1E-02	3,17
O35945	Aldehyde dehydrogenase, cytosolic 1	Aldh1a7	2,4E-02	2,92
P62082	40S ribosomal protein S7	Rps7	2,2E-02	2,76
Q78KK3	Solute carrier family 22 member 18	Slc22a18	3,5E-02	2,54
P62821	Ras-related protein Rab-1A;Ras-related protein Rab-1B	Rab1A;Rab1b	1,5E-02	2,37
Q8VCH0	3-ketoacyl-CoA thiolase B, peroxisomal	Acaa1b	2,0E-02	2,29
Q9JHW2	Omega-amidase NIT2	Nit2	3,5E-02	0,48
P55264	Adenosine kinase	Adk	1,2E-03	0,47
P24527	Leukotriene A-4 hydrolase	Lta4h	1,4E-02	0,44
Q02248	Catenin beta-1	Ctnnb1	2,5E-02	0,42
P06745	Glucose-6-phosphate isomerase	Gpi	1,1E-02	0,42
Q99L88	Beta-1-syntrophin	Sntb1	1,7E-02	0,41
P68134	Actin, alpha skeletal muscle;Actin, alpha cardiac muscle 1;Actin, gamma-enteric smooth muscle;Actin, aortic smooth muscle	Acta1;Actc1;Actg2;Acta2	3,7E-02	0,37
Q99K01	Pyridoxal-dependent decarboxylase domain-containing protein 1	Pdxdc1	5,9E-03	0,34
Q8BUV3	Gephyrin	Gphn	1,2E-03	0,34
Q61694	3 beta-hydroxysteroid dehydrogenase type 5;3 beta-hydroxysteroid dehydrogenase type 4	Hsd3b5;Hsd3b4	1,2E-02	0,06
